# The impact of retirement on health: quasi-experimental methods using administrative data

**DOI:** 10.1186/s12913-016-1318-5

**Published:** 2016-02-19

**Authors:** Elizabeth Mokyr Horner, Mark R. Cullen

**Affiliations:** American Institutes for Research, 2800 Campus Dr. Suite 200, San Mateo, CA 94330 USA; Stanford University School of Medicine, Population Health Sciences, MSOB 1265 Welch Road, Stanford, CA 94305 USA

**Keywords:** Retirement, Physical health, Quasi-experimental, Claims data

## Abstract

**Background:**

Is retirement good or bad for health? Disentangling causality is difficult. Much of the previous quasi-experimental research on the effect of health on retirement used self-reported health and relied upon discontinuities in *public* retirement incentives across Europe. The current study investigated the effect of retirement on health by exploiting discontinuities in *private* retirement incentives to test the effect of retirement on health using a quasi-experimental study design.

**Methods:**

Secondary data (1997–2009) on a cohort of male manufacturing workers in a United States setting. Health status was determined using claims data from private insurance and Medicare. Analyses used employer-based administrative and claims data and claim data from Medicare.

**Results:**

Widely used selection on observables models overstate the negative impact of retirement due to the endogeneity of the decision to retire. In addition, health status as measured by administrative claims data provide some advantages over the more commonly used survey items. Using an instrument and administrative health records, we find null to positive effects from retirement on all fronts, with a possible exception of increased risk for diabetes.

**Conclusions:**

This study provides evidence that retirement is not detrimental and may be beneficial to health for a sample of manufacturing workers. In addition, it supports previous research indicating that quasi-experimental methodologies are necessary to evaluate the relationship between retirement and health, as any selection on observable model will overstate the negative relationship of retirement on health. Further, it provides a model for how such research could be implemented in countries like the United States that do not have a strong public pension program. Finally, it demonstrates that such research need-not rely upon survey data, which has certain shortcomings and is not always available for homogenous samples.

## Background

### Introduction

What is the relationship between retirement and health? This is a question of importance to individuals, actuaries, businesses, and governments [1]. Lifespans are increasing and retirement norms have not adapted; people are now spending a larger proportion of their lives retired [2]. Theoretically, it is possible that retirement is good for health, as physical and psychological stress may be reduced [3–5]; others argue continued work can be protective [6–8]. Of course, it is possible that retirement has heterogeneous effects on different populations [9, 10], or over time [11]. Investigating the health benefits or consequences of retirement decisions is one of the first steps in addressing the mounting costs of supporting an aging population.

However, health clearly impacts the retirement decision, and disentangling the directionality of the relationship is complicated. One common method uses longitudinal data and controls for pre-retirement health and characteristics. Other researchers have employed quasi-experimental methodologies, exploiting discontinuities in retirement incentives, in other words, discrete changes (usually age related) in the incentives individuals face to retire. These studies paint a more favorable picture of retirement. The vast majority of previous research on retirement and health has relied on self-reported measures of health from surveys.

### Methodological shortcomings of previous research

Despite significant research on the topic, a recent systematic review of research using longitudinal data and person-level fixed effects [10] found “no univocal effect [of retirement] on perceived general health and physical health” (p. 8). Indeed, as the authors suggest, studies find a wide variety of effects with little consensus. Some research has found that retirement is detrimental to physical and mental health [e.g., [6, 9]], while others have suggested potentially positive effects [12], particularly for mental health [e.g., [2, 4, 13, 14]].

#### Problem 1: dynamic endogeneity of retirement decisions

One likely source of the variety of results is caused by the fact that people choose to retire, often for reasons we cannot directly observe. Any Selection on Observables (SOO) Models, even those controlling for baseline health, can only account for what is known prior to the retirement decision. In other words, this methodology cannot account for the *dynamic* relationship between health and retirement; for example, someone who is healthy at age 50 may begin to experience symptoms of a chronic disease right around the time he or she decides to retire at age 64, and may get an official diagnosis immediately thereafter. Indeed, researchers have found that health considerations motivate retirement decisions [15, 16], and may be even more important than economic factors [17, 18].

#### Problem 2: lack of external validity to sample of United States workers

SOOs have limitations as described above, and randomization (experimental design) is not feasible; as such, some researchers have relied on post-hoc randomization (quasi-experimental design). This research has taken advantage of public pension programs in Europe, which have been shown to increase retirement [19–21]. These quasi-experimental studies have found neutral to positive effects for physical health [12, 22, 23].

The quasi-experimental study designs described above may be the best way to look for causal relationship between retirement and health. However, the United States have a relatively weak public pension program, primarily set up as a social safety net, that does little to alter retirement behavior, particularly among better paid workers [24]. There has been limited previous research using a quasi-experimental design on a United States sample, and the studies that exist have had to bolster an instrument relying on public pensions. For example, Charles (2004) included in his instrument changes in the pension system occurring in the early 1980s [24]. Using more recent data, Neuman [23] included self-reports of whether the individual has become eligible for a private pension. This work builds these previous studies; we do not rely on self-reports for whether individuals are eligible for their pensions and we limit our samples to workers offered pensions.

#### Problem 3: self-report may not be ideal for health research among the aged

These projects (and indeed, most studies on health in retirement) have relied heavily on self-reported health and diagnoses [10], likely in part due to the widespread availability of survey data on the older individuals, i.e., the Health and Retirement Survey (HRS) in the US, the English Longitudinal Survey of Ageing (ELSA), the Survey of Health and Retirement in Europe (SHARE), the Mexican Health and Aging Study (MHAS), and the Chinese Health and Retirement Survey (CHARLS), and many others. Though few exceptions exist (e.g., using prescriptions: Oksanen et al., [25]; using death records: Bound & Waidmann, [22]), they are few and offer insights on a narrow aspect of health.

Clearly there are some major advantages to survey data; for example, it is possible to get a window into one’s subjective experience of their own health. Global self-reported health (e.g., “Would you say your health is excellent, very good, good, fair, or poor?”) is a purposefully subjective and global measure. In addition, it is possible to ask people about their ability to complete Activities of Daily Living (ADL), such as their ability to self-feed. However it is difficult to disentangle how much of these self-perceived variables are caused by actual physical health versus other drivers, such as mental health, mood, and affect.

To triangulate a more objective measure of health, survey participants have often been asked whether they have been diagnosed with specific diseases (e.g., “Has a doctor ever told you that you have *diabetes*?”) or if they can complete basic Activities of Daily Living (e.g., “Without assistance are you able to *dress*?”). These questions were designed to be comparable across respondents and be specific enough to “constrain the likelihood that respondents rationalize their own behavior through their answers” [26]. However, there is some evidence that these responses are not always valid when compared with physician records [26, 27].

Importantly, these measures are quite weak when very precise onset dates are needed, in part due to age heaping, a phenomenon that has been observed in census data wherein older individuals over-report “round” ages such as 60, 65, 70 etc. It should be noted that some of the apparent age heaping may be due to age-based screening, but it is impossible to know how much. The self-reported ages interpolated from reported onset dates from the Survey of Health and Retirement in Europe (SHARE) are reported in Appendix A, indicating substantial age heaping in these data.

### This project

The current study proposes a new potential protocol for addressing the research question: How does retirement affect health? The current project utilizes a set of health claims from a sample of American manufacturing workers to investigate the relationship between retirement and health using an instrumental variables methodology.

Here, we demonstrate the use of health claims from individuals’ work-lives integrated with Medicare claims to provide insight into retirement health, exploiting discontinuities in a private retirement incentive plan. By exploiting exogenous variation in *private* pensions, we are able to use a similar methodology to those who are studying the effects of retirement in Europe, where there are strong public incentives to retire. Further, by using health claims rather than self-report we have much greater precision around onset dates as described in Problem 3 above. This protocol will become increasingly useful as linkable claims data become more common; for example, Stanford University will become a data repository for a wide array of linkable claims data in the near future [28].

In sum, this paper has two aims: 1) to explore the plausibly causal relationship between retirement and health in a sample of manufacturing workers; and 2) to demonstrate how this quasi-experimental methodology using employer-based health insurance claims data and administrative data including retirement incentives can provide insight into the effect of retirement on health.

## Methods

### Sample

Data were obtained for hourly and salaried employees at a geographically diverse aluminum production company who worked a day or longer between January 1, 1996 and December 31, 2009. The individuals’ administrative data were linked to their private and public health claims. In this sample, the majority (69 %) remained insured after they retired, due in part to the low premiums their unions have negotiated.

It should be mentioned that these workers were working in very physically demanding jobs. Some studies have found that individuals who were in more physically or psychologically demanding jobs had a differentially high benefit from retirement [9, 10], while other have failed to find any difference between the effects of retirement on blue- and white-collar workers [3, 29–32]. Regardless, this is an important characteristic of this sample to consider.

This sample was limited to men born (1932–1944), as they reached retirement age early enough to be observed for several years post-retirement. In addition, the men facing the incentives used as an instrument were all unionized hourly workers facing a homogenous retirement incentive (*N* = 1,836). Some data were not available because a match was not found using Social Security numbers, dates of birth, and full names. Other individuals’ data were not available because they chose Medicare Advantage Plan or opted out of Medicare Plan B (ambulatory care coverage). The sample was limited to individuals for whom Medicare data fully available (*N* = 1,076). In addition, we excluded individuals who had an acute health crisis during the window, as indicated by a death prior to age 70 or a hospitalization greater than 10 days because they might have experienced an idiosyncratic health catastrophe in their mid60s that could bias our findings (*N* = 1,008).

For some analyses, we further limit our sample to individuals for whom we have continuous data (*N* = 659). This excludes men who did not purchase health insurance during all of the “gap” (63–64) and are thus unobserved for some portion of that time. It is not known why some individuals did not purchase insurance during this gap; it is possible that they purchased a less-extensive catastrophic insurance, that they obtained coverage through a spouse, or that they chose to be uninsured. It is possible that some of these individuals did not purchase insurance during the gap because they were averse to obtaining health care. Regardless, for the purposes of this study, we only have claims data for individuals while they are insured through their employment based private insurance or through Medicare.

Administrative data are sometimes missing for a variety of idiosyncratic reasons and therefore the exact number of individuals included in different analyses varies. This is discussed in greater length in the Conclusion section under Study Limitations.

### Key variables

Health status was determined using International Classification of Disease (ICD-9) codes for the following diseases: hypertension, diabetes, asthma/COPD, arthritis, and major depression. These ailments were chosen because of the relatively high rates in this population, as well as the fact that these diseases greatly may impact quality of life but are not generally measurable as outcome variables when mortality data are used because they are not the cause of death. Some other conditions of interest, such as cancer, could not be examined because of their rarity pre-retirement and limits of data availability in this still relatively young cohort all actively working at least as recently as 1996.

Prevalence of these diseases was determined as follows: if an individual has had the relevant ICD-9 codes occurring in two outpatient visits within one year or one inpatient visit within any of the years for which they have employer-based health insurance, they were considered to have the disease. This algorithm has been validated for these diseases with this population [e.g., [1, 33–35]]. Prevalence was considered as the outcome variable of choice because these diseases can be controlled but are frequently not curable. Health utilization (number of inpatient and face-to-face outpatient visits) was also studied.

Where indicated, *risk scores* at age 61 were included as a control. Risk scores are a metric forecasting future healthcare consumption as a function of previous utilization, age, ailments, and a variety of other individual characteristics. The risk scores used for this paper were created using software produced by Verisk Health, which implements the Diagnostic Cost Group Hierarchical Condition Category (DxCG-HCC) classification model. Although the actual algorithm for risk score is a black-box, there is some recent research validating these scores as good predictors of upcoming health problems [36].

### Summary statistics

**Fig. 1:**
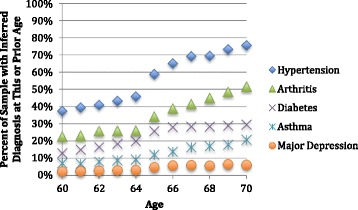
Cross-sectional disease prevalence (1996–2009)

Table 1 provides summary statistics on the sample. As can be seen, this is a relatively homogenous sample. Disease prevalence by age is provided in Fig. 1. Although the proportion of the sample with these diseases may at first glance appear high, these rates have been confirmed with biometric test results for hypertension (blood pressure tests) and asthma/COPD (spirometry tests) on this cohort [1, 37]. In addition, an increase in diagnoses was seen at age 65 (Fig. 1); these are likely not new ailments but rather prevalent illnesses that were captured by the high-level “Welcome to Medicare” Evaluation and Management visit which includes a more thorough health history [1, 38]. Because this increase in diagnosis occurs at age 65 rather than age 62 (when individuals retire), this is not biasing for this sample.

**Table 1:** Summary statistics, administrative data (1996–2009)

	Mean (SD)	Range
Year	2001.3 (3.5)	1996-2009
Year of birth	1940.2 (2.3)	1932-1944
Age Retired	62.4 (2.5)	54-70
Number of Plants	30	
Number of People, Total	1,841	
Number of People, Sufficient Data Available	1,008	
Number of People, Observed Continuously	659	
% Person-years retired	31 %	
% People insured while working	83.1 %	
% People insured while retired, pre age 65	69.1 %	
% People with supplemental insurance age 65+	26.4 %	
% Unionized Men Retired by Age 61	15.5 %	
% Unionized Men Retired by Age 62	26.1 %	% Δ = 68 %
% Unionized Men Retired by Age 63	51.0 %	% Δ = 95 %
% Unionized Men Retired by Age 64	69.3 %	% Δ = 36 %
% Unionized Men Retired by Age 65	78.4 %	% Δ = 13 %

### Empirical framework

Previous quasi-experimental research has been done on European samples exploiting differences in the *public* incentives to retire, namely the discontinuities in retirement incentives that occur at the early and normal retirement ages [2, 12, 22, 39]. Note that like the current study, many of the previous studies focused exclusively on men, finding that women in this cohort are more weakly connected to the workforce in general, more likely to retire early, and less responsive to financial incentives to retire [12, 22–24].

Our project exploited differences in the *private* incentives to retire, specifically the availability of a pension among unionized hourly workers at a set of United States based manufacturing plants. These workers received a generous pension once they reached age 62; thus the rapid increase of retirement occurring as these individuals become eligible for their pensions is unsurprising. Importantly, these workers have a homogeneous defined-benefit pension plan, and access to health coverage and pension plan are both universal in this sample.

Thus, this study utilizes an Instrumental Variables (IV) design. As always, the IV methodology can be described as occurring in two stages. Specifically consider:

- Stage 1: Retired_i_ = a_1_Instr_i_ + a_2_AgePoly_i_ + a_3_∑Plant_P_ + v_i_
- Stage 2: $$ {\mathrm{DepVar}}_{\mathrm{i}} = {\mathrm{b}}_1{\overbrace{\mathrm{R}}}_{\mathrm{i}} + {\mathrm{b}}_4{\mathrm{AgePoly}}_{\mathrm{i}} + {\mathrm{b}}_3\sum {\mathrm{P}\mathrm{lant}}_{\mathrm{P}} + {\mathrm{v}}_{\mathrm{i}} $$

Where Instr_i_ is a dummy variable reflecting whether the individual has reached the age that makes him eligible for the highest level of pension as per his union agreement (age 62). Retirement was estimated as a function of all of the included explanatory variables as well as these additional instruments. The models employed account for age flexibly, which should control for any smooth, age-related trends in health. Thus, the results will reveal whether there is a discrete, non-monotonic change in health in the years surrounding these discontinuities as a function of retirement. In addition, controls for which plant the individual worked were also included.

Stage 1 reveals ${\overbrace{\mathrm{R}}}_{\mathrm{i}}$, an estimation of retirement that is exogenous to observed and unobserved individual-level characteristics; this estimation was used instead of the individual’s true retirement status providing an estimation of the effect of retirement that is exogenous to individual choice.

A good instrument has two key requirements: 1) the instruments must be related to the endogenous variable it is being used to estimate; and 2) the instruments must *only* affect the outcome variable through the endogenous variable that it is being used to estimate. It is straightforward to show that discontinuities in retirement incentives passed the first criteria for instruments. We were able to show that turning 62 had a large effect on retirement decisions in two ways. First, the proportion retired by age is depicted in Table 1—notice that the proportion retired jumps from 13.9 – 51 % between ages 61 and 63, thus providing a strong instrument for retirement: whether the individual has reached pension eligibility at age 62. Second, it is necessary make sure that all instruments have F-statistics above the cutoff for sufficiently strong instruments (10.00[40]); our F-statistics are presented for all IV models and are consistently above this cutoff.

Passing the second requirement is more complicated [40]. Because disease diagnoses are a function of doctor visits, there are a few possible pathways. For example, if going to the doctor became relatively more expensive at retirement, this would result in less medical care, therefore creating the illusion people were *healthier* due to fewer diagnoses. However, this is unlikely to be a problem in our data, because this population encountered its retirement incentives relatively early (prior to Medicare), and the insurance they received between retiring and age 65 was the same insurance that they had during their working lives.

On the other hand, if going to the doctor became relatively less expensive at the incentive kinks, perhaps because retired workers have lower opportunity costs for going to the doctor, individuals might appear *less* healthy due to an increase of diagnoses. This is a legitimate weakness of using an IV methodology for health outcomes; healthcare utilization may increase at retirement making individuals appear to have more chronic diseases. As such, it is important to evaluate whether there is a change in the number inpatient and outpatient visits for this sample as a function of estimated retirement. If there is an increase in visits, then these results should be seen as an upper bound for the negative effect of retirement on health.

### Additional specification notes

Because the instruments allow models to behave as post-exposure randomization, omitted demographic variables should not be a source of bias [41]. Thus, although there are many variables that could contribute to the retirement decision, to health, or to the size and direction of the relationship between them, omission of these variables should not bias our estimates. The models presented are Linear Probability Models (LPM). While binary outcome variables are often modeled using logistic regressions, there are several advantages to using LPMs when using a two-stage-model. First, with plausibly causal models, linear models produce clear marginal differences *even* when the outcome variables are binary [40]. Second, LPM are convenient, computationally tractable, and may have less bias than alternatives [42]. Finally, they have the added advantage of being easy to interpret; for example a coefficient of .015 with a binary outcome can be interpreted as a 1.5 % change for every one-unit change in an explanatory variable. The sample was limited to men 55–70 years of age. Finally, the standard errors were clustered by plant. Because the number of clusters was quite small, this would have the effect of attenuating findings [43].

The Stanford University Institutional Review Board approved this study’s protocol, invoking the epidemiologic exemption waiving the requirement for individual consent.

## Results

Table 2 depicts the results for individuals for whom continuous health data are available. The SOO models overstate utilization associated with retirement and the coefficients on health outcomes tend to be positive, though largely insignificant, with the exception of diabetes—this may be in part due to the small sample size. Include risk scores at age 61, a strong control for observable underlying health, attenuates the coefficients. Yet, the IV-model still reveals that the both SOO models overstate the negative impact of retirement. This is in keeping with previous research on retirement and health, reflecting that SOO models will overstate both the negative impact of retirement on health and the healthcare utilization caused by retirement.

**Table 2:** Effect of retirement on health for only continuously insured individuals, administrative data (1996–2009)

Coefficient [Standard Error]	Row Mean	SOO	SOO & Risk	IV
Hypertension	44 %	0.0613	0.0453	−0.0891
		[0.0517]	[0.0463]	[0.0780]
Diabetes	17 %	0.0846**	0.0846*	0.00563
		[0.0319]	[0.0381]	[0.0477]
Asthma	8 %	0.0157	0.00029	−0.0729***
		[0.0137]	[0.0148]	[0.0222]
Arthritis	26 %	0.0742	0.0689	−0.0727
		[0.0482]	[0.0580]	[0.0557]
Major Depression	3 %	0.00967	0.0115	−0.00365
		[0.0126]	[0.0152]	[0.0199]
Inpatient Visits	0.11	0.0493**	0.0368	−0.0826
		[0.0211]	[0.0220]	[0.138]
Outpatient Visits	4.84	1.688***	1.456***	0.0899
		[0.206]	[0.257]	[0.422]
N	659	659	524	659
Excluded Instrument F-Stat		N/A	N/A	22.49

***p < 0.01, ** *p* < 0.05, * *p* < 0.1

Results presented are derived from fourteen independent regression models using administrative data. For hypertension, diabetes, asthma, arthritis, and major depression, the outcome variable was whether the individual had—this year, or previously—received a diagnosis for this illness, using the algorithm described in the Data section. For inpatient and outpatient visits, the outcome variable was the number of face-to-face, unique visits of that type

The first column reports the coefficients on *retirement* using traditional selection on observables models. The second column reports the coefficients on *retirement* where retirement was estimated using instrumental variables as described in the Empirical Framework section. This sample consisted of continuously insured unionized men ages 55–70. Controls for plant and an age polynomial were included

In our sample, we find a reduction in asthma at retirement. Because the sample is relatively small (*N* = 658), representing a very specific population and the point estimates are altered by sample selection, the specific rates should not be over interpreted. For example, notice that it appears that asthma decreases by about 7 % at retirement. Although the confidence interval is wide, this is a somewhat implausible magnitude given the row mean. Given the small sample size, the size of coefficients should be interpreted cautiously as suggestive rather than definitive.

Table 3 includes all individuals for which we had pre and post retirement data, including individuals who retired at age 62 and did not purchase health insurance during all of the “gap” (63–64) and are thus unobserved for some portion of that time. Notice that the results are rather similar, and the coefficient on asthma is somewhat more believable. This suggests that with an even larger sample, we would be afforded greater precision on a likely smaller point estimate for the impact of retirement on asthma.

**Table 3:** Effect of retirement on health for all unionized workers, administrative data (1996–2011)

Coefficient [Standard Error]	Row Mean	SOO	SOO & Risk	IV
Hypertension	50 %	0.0794	0.0661	−0.00174
		[0.0441]	[0.0422]	[0.0611]
Diabetes	20 %	0.0844**	0.0890**	0.0613
		[0.0282]	[0.0325]	[0.0384]
Asthma	10 %	0.0207	0.0112	−0.0405**
		[0.0119]	[0.0135]	[0.0177]
Arthritis	29 %	0.0839*	0.0839	−0.0187
		[0.0445]	[0.0542]	[0.0658]
Major Depression	4 %	0.0122	0.0161	0.00148
		[0.0113]	[0.0115]	[0.0180]
Inpatient Visits	0.11	0.0745***	0.0545**	0.0228
		[0.0179]	[0.0207]	[0.123]
Outpatient Visits	4.83	1.784***	1.680***	0.746
		[0.173]	[0.176]	[0.485]
N	1,008	1,008	706	1,008
Excluded Instrument F-Stat		N/A	N/A	68.22

***p < 0.01, ** *p* < 0.05, * *p* < 0.1

Results presented are derived from fourteen independent regression models using administrative data. Disease prevalence outcome variables reflect whether the individual had —this year, or previously—received a diagnosis for this illness, using the algorithm described in the Data section. For inpatient and outpatient visits, the outcome variables were the number of face-to-face, unique visits of that type

The first column presents the row means, or cross-sectional prevalence, in this sample. The second column reports the coefficients on *retirement* using traditional selection on observables models. The third column reports the coefficients on *retirement* using traditional selection on observables models, but including a control for risk-score at age 61. The fourth column reports the coefficients on *retirement* where retirement is estimated using instrumental variables as described in the Empirical Framework section. The sample consisted of unionized men ages 55–70

Standard errors were clustered by country plant. Controls for plant and an age polynomial were included

Note that the F-statistic is quite high for both samples (22.49 and 68.22). This may be because this sample is fairly homogeneous and faces strong incentives to retire. It is also possible this is because there is very little measurement error; retirement dates are known with some certainty.

One notable difference between this project on previous literature is that no effect was found on mental health; previous research using IVs have found a positive effect from retirement [e.g., [2, 14, 24]]. This may reflect a limitation of these more objective measures of health, since the bar for depression using claims data is structurally set high—data incorporating antidepressant prescriptions, which were not available on all of this sample, has found significantly higher levels of depression [34]. Individuals with borderline depression who did not obtain medical care for their mood were not considered depressed, and those individuals who obtained prescriptions for their depression but who did not receive the ICD-9 codes would have been missed.

## Discussion

### Primary findings

First and foremost, this study finds a neutral effect of retirement on most measured aspects of health and healthcare utilization, and a reduction in asthma. One possible exception is that retirement may cause increased risk for diabetes. In addition, this paper provides evidence that quasi-experimental study designs are important to the study of the effects of retirement. Further, it documents the importance of administrative data, like claims, in tracking the trajectory of chronic disease onset around retirement, as self-reports of onset dates are likely biased (see Appendix A). Finally, it proposes a methodology to examine the relationship between health and retirement within a United States context, namely, to link administrative data to health claims data and exploit discontinuities in *private* pensions.

This study offers several major lessons for researchers. First, any SOO study design is going to overstate the negative impact of retirement due to underlying differences in the population. Due to the dynamic nature of the retirement decision, controls for static individual level characteristics such as individual-level fixed effects or composite scores for underlying health will not necessarily solve this problem, namely that some of the health-related reasons for individuals to choose retirement early may be of recent origin—not fixed effects—and/or totally unobserved.

Second, this paper provides some general observations about the strengths and weaknesses of these types of more objective measures of health when using an instrumental variables approach. As shown in the Appendix, individuals’ accounts of diagnosis age are often unreliable and tended to coincide with the kinks in retirement incentives, likely creating biased outcomes. Administrative data may address these weaknesses.

Finally, though there may not be adequate public sources of exogenous variation retirement behavior in the United States, there may well be sources within the private sector. Researchers who find such instruments can link administrative data with claims data from the worklife and from Medicare in order to gain access to questions around health and retirement. Administrative data from individuals’ work-life allows for powerful instruments to study retirement.

### Study limitations

This study does have important limitations. First, it is important to carefully interpret results. Because quasi-experimental design generates Local Average Treatment Effects rather than an average treatment effect, they should not be considered prescriptive, particularly at different retirement ages or with different samples. In addition, this is a rather specific sample of hourly, unionized, manufacturing workers. Such jobs resemble many occupations, but are not necessarily “typical” of all work. However, idiosyncratic populations are likely to be the norm for such retirement research in the United States; samples facing similar enough and strong enough retirement incentives to generate a quasi-experiment will be specific, small, and for the foreseeable future, largely male.

Second, this study provided a proof of concept on a small sample. Due to the small sample size, it is not possible to know if some results were null due to adequate power. Future studies on this population will benefit from a growing relevant sample, and similar research on other populations should take care that sample size is likely to be a frequent problem. Second, perhaps due in part to the small sample size, our results were not particularly robust to specification; however, the general conclusions were the same across specifications. Third, using claims instead of direct diagnostic data such as might be obtained from electronic medical records or by examination means that results should be interpreted with care.

Finally, administrative data can be difficult to work with. These data were relatively complete but did contain missing elements for a subpopulation. Some individuals lacked a risk score, and others spent some time between age 60 and age 65 with a different primary insurance and have a year or more of missing claims data. Little can be done about this, and perhaps future researchers with larger samples may be able to afford to drop individuals with any missing data. In addition, it is important to be careful that findings are not artefacts of the data; if, for example, a sample’s private pensions occur very close to age 65, an increase of diagnoses due to the “Welcome to Medicare” will create specious results.

## Conclusions

The linkage of work-life administrative data with retirement health is still a poorly tapped resource [1]. Researchers wishing to examine health outcomes of retirement, particularly in the United States, should consider forming such linkages across many firms and industries. This may reveal heterogeneous treatment effects by job type and by the timing of incentives. As lifespans lengthen, these questions are becoming increasingly important.

## Appendix A: Evidence of age heaping

In the 2011 Survey of Health and Retirement in Europe (SHARE), individuals were asked a set of questions: “At what age were you first told you had *XX?*” The number of people who stated they were first diagnosed with a disease at that age is presented. As can be seen, there is substantial over-reporting of diagnoses at ages that are multiples of five. When onset dates are important, self-reported histories may not be ideal.

**Table 4:** Number of individuals reporting age first diagnosed, SHARE Survey (2011)

Age	Hypertension	Cholesterol	Diabetes	Asthma	Arthritis
49	236	138	65	31	111
*50*	*1682*	*974*	*413*	*169*	*950*
51	255	158	88	48	143
52	412	270	137	40	222
53	329	216	96	45	151
54	299	187	121	40	174
*55*	*1000*	*615*	*303*	*105*	*561*
56	318	208	139	46	169
57	320	209	116	47	166
58	396	253	128	39	228
59	225	174	93	36	108
*60*	*1082*	*708*	*359*	*131*	*663*
61	204	121	73	21	63

## Abbreviations

CHARLES: Chinese health and retirement survey
COPD: Chronic obstructive pulmonary disease
ELSA: English longitudinal survey of ageing
HRS: Health and retirement survey
ICD9: International classification of disease
IV: Instrumental variables
LPM: Linear probability model
MHAS: Mexican health and aging study
SHARE: Study of health and retirement in europe
SOO: Selection on observables
